# Overview of the BioCreative III Workshop

**DOI:** 10.1186/1471-2105-12-S8-S1

**Published:** 2011-10-03

**Authors:** Cecilia N Arighi, Zhiyong Lu, Martin Krallinger, Kevin B Cohen, W John Wilbur, Alfonso Valencia, Lynette Hirschman, Cathy H Wu

**Affiliations:** 1 Center for Bioinformatics and Computational Biology, University of Delaware, Newark, DE, USA; 2 National Center for Biotechnology Information, National Library of Medicine, Bethesda, MD, USA; 3 Structural and Computational Biology Group, Spanish National Cancer Research Centre, Madrid, Spain; 4 Center for Computational Pharmacology, University of Colorado Denver School of Medicine, Aurora, CO, USA; 5 The MITRE Corporation, Bedford, MA, USA

## Abstract

**Background:**

The overall goal of the BioCreative Workshops is to promote the development of text mining and text processing tools which are useful to the communities of researchers and database curators in the biological sciences. To this end BioCreative I was held in 2004, BioCreative II in 2007, and BioCreative II.5 in 2009. Each of these workshops involved humanly annotated test data for several basic tasks in text mining applied to the biomedical literature. Participants in the workshops were invited to compete in the tasks by constructing software systems to perform the tasks automatically and were given scores based on their performance. The results of these workshops have benefited the community in several ways. They have 1) provided evidence for the most effective methods currently available to solve specific problems; 2) revealed the current state of the art for performance on those problems; 3) and provided gold standard data and results on that data by which future advances can be gauged. This special issue contains overview papers for the three tasks of BioCreative III.

**Results:**

The BioCreative III Workshop was held in September of 2010 and continued the tradition of a challenge evaluation on several tasks judged basic to effective text mining in biology, including a gene normalization (GN) task and two protein-protein interaction (PPI) tasks. In total the Workshop involved the work of twenty-three teams. Thirteen teams participated in the GN task which required the assignment of EntrezGene IDs to all named genes in full text papers without any species information being provided to a system. Ten teams participated in the PPI article classification task (ACT) requiring a system to classify and rank a PubMed^®^ record as belonging to an article either having or not having “PPI relevant” information. Eight teams participated in the PPI interaction method task (IMT) where systems were given full text documents and were required to extract the experimental methods used to establish PPIs and a text segment supporting each such method. Gold standard data was compiled for each of these tasks and participants competed in developing systems to perform the tasks automatically.

BioCreative III also introduced a new interactive task (IAT), run as a demonstration task. The goal was to develop an interactive system to facilitate a user’s annotation of the unique database identifiers for all the genes appearing in an article. This task included ranking genes by importance (based preferably on the amount of described experimental information regarding genes). There was also an optional task to assist the user in finding the most relevant articles about a given gene. For BioCreative III, a user advisory group (UAG) was assembled and played an important role 1) in producing some of the gold standard annotations for the GN task, 2) in critiquing IAT systems, and 3) in providing guidance for a future more rigorous evaluation of IAT systems. Six teams participated in the IAT demonstration task and received feedback on their systems from the UAG group. Besides innovations in the GN and PPI tasks making them more realistic and practical and the introduction of the IAT task, discussions were begun on community data standards to promote interoperability and on user requirements and evaluation metrics to address utility and usability of systems.

**Conclusions:**

In this paper we give a brief history of the BioCreative Workshops and how they relate to other text mining competitions in biology. This is followed by a synopsis of the three tasks GN, PPI, and IAT in BioCreative III with figures for best participant performance on the GN and PPI tasks. These results are discussed and compared with results from previous BioCreative Workshops and we conclude that the best performing systems for GN, PPI-ACT and PPI-IMT in realistic settings are not sufficient for fully automatic use. This provides evidence for the importance of interactive systems and we present our vision of how best to construct an interactive system for a GN or PPI like task in the remainder of the paper.

## Background

There is a long history of shared or competitive tasks in the applied computational sciences. The Message Understanding Conferences (MUC) began in 1987 and involved seven conferences over a ten year period. The focus of the MUCs was extraction of different categories of events from newswire. These conferences quickly established a general methodology of providing gold standard humanly judged data, and requiring a common result format and common evaluation measures for all participants[1]. This approach has been adopted in many other areas and has generally fostered performance improvements. The Text REtrieval Conferences (TREC) began in 1992 and have been held yearly since. They follow the general methodology of the MUC conferences, but focus on information retrieval from large textual databases (http://trec.nist.gov/pubs.html). In 1994 the Critical Assessment of Techniques for Protein Structure Prediction (CASP) began as a biennial evaluation of protein structure prediction methodologies (http://predictioncenter.org/index.cgi). Since these beginnings many other community wide competitions have appeared with the objective of fostering improved performance in different areas of computational science [2].

### Text mining in biology

Text mining in biology has emerged as an important area of research for two main reasons. First, many fields within biology, especially molecular biology, exist in a large body of published literature which describes findings, methods and associated experimental evidence and the sheer volume of this data makes some kind of organization into databases necessary to improve accessibility; second, biologists have become increasingly dependent on access to “computable” biological data available in public biological databases, often derived via expert curation from unstructured text in the biological literature. Biology therefore presents data-rich natural language resources expressed in a distinctive sublanguage of English [2,3], with extensive lexical community resources, including taxonomies, ontologies and controlled vocabularies used in the structured resources. The biologists’ need for access to information in free text, and the richness of resources for natural language processing, have led to a number of shared competitions in biomedical text mining. The KDD-CUP challenge for 2002 involved a task dealing with FlyBase [4]. Contestants were given papers and the genes occurring in those papers and were asked to determine which papers qualified for curation in FlyBase and if a paper had experimental evidence of gene expression to determine which genes had corresponding gene products. This was the precursor to the BioCreative Workshops (2004-2010) and largely set the focus on assisting the curator and using existing curated data as the basis for gold standard training and test data. The interaction article subtask (IAS) or article classification task (ACT) of BioCreative II & II.5 is similar to the KDD-CUP challenge of 2002. The theme of assisting the curator has continued to the present.

These activities have been augmented by a number of other text mining efforts in the biology domain. The TREC Genomics track [5] (2003-2007) focused on information retrieval and question answering for biologically relevant queries. The Joint Workshop on Natural Language Processing in Biomedicine and its Applications (JNLPBA-2004) [6] was based on the GENIA corpus [7] and had as its goal tagging of all instances of proteins, DNA, RNA, cell lines and cell types occurring in a set of MEDLINE abstracts of the same kind as those in the GENIA corpus. The Learning Language in Logic Workshop (LLL05) focused on learning how to recognize genic interactions involving *Bacillus subtilis* genes described in MEDLINE abstracts [8]. The BioNLP 2009 shared task [9] required the extraction of bio-molecular events from GENIA documents (MEDLINE abstracts) where genes/proteins were pre-annotated in the text. The GENIA corpus is restricted to MEDLINE records indexed with the three MeSH terms, *human*, *blood cell*, and *transcription factor*[6]. The CALBC (Collaborative Annotation of a Large Biomedical Corpus) Challenge began in 2009 and is ongoing with a community wide effort to annotate multiple semantic types over a set of approximately 150,000 MEDLINE abstracts in the area of immunology [10,11]. The goal is to harmonize the output of multiple algorithms designed by different research groups to produce a silver standard set of annotations which will be made available to the text mining community. All of these tasks have focused on some aspect of text mining in biology, often with a restricted subject matter and largely with a goal of improved natural language processing in support of biomedical applications.

There have been similarities between the other tasks just described and the BioCreative Workshops, as is evident in Table 1. The labeling task of JNLPBA-2004 is similar to the gene mention (GM) task of BioCreative I & II and the LLL05 task bears some similarity to the protein-protein interaction extraction tasks of BioCreative II & II.5, though the latter were focused on full text articles. Both tasks are somewhat coarser grained than the BioNLP’09 Shared task. The CALBC Challenge includes the GM task of BioCreative I & II. However, the BioCreative Workshops, from their inception, have had the broader goal of promoting the development of practical text mining tools for database curators and the users of textual data in the field of biology [12]. This has influenced the choice of tasks so that, where appropriate data has been available, tasks have dealt with full text. Also species have only been restricted in the case of the GN tasks for BioCreative I & II where it was done largely for reasons of tractability. Another aspect of practical importance, but not reflected in Table 1, is the development of a BioCreative MetaServer [13] which makes available to database curators and those interested in text mining, the annotations produced by a number of systems that participated in BioCreative II. In the same spirit teams were encouraged to set up servers for online testing of their systems in BioCreative II.5 [14]. Indeed if there are themes that set the BioCreative effort apart from other similar efforts, we believe these are the emphasis placed on the development of practical text mining systems for biology and the links to ongoing biological curation and annotation activities as the source for training and test (gold standard) sets.

**Table 1:** Tasks performed by participants in the four BioCreative Workshops held to date. Abbreviations are defined as follows, interacting protein normalization task (INT), interaction article subtask (IAS) or article classification task (ACT), interaction methods subtask (IMS) or task (IMT), interaction pairs subtask (IPS) or task (IPT), interaction sentence subtask (ISS).

Workshop Task	BioCr I 2004	BioCr II 2007	BioCr II.5 2009	BioCr III 2010
Gene Mention (GM)	sentences abstract	sentences abstract		

Gene Normalization (GN)	Fly, Yeast, Mouse abstract	Human abstract	INT(proteins), full text	full text

Gene Ontology (GO) codes	Evidence full text			
	
	Codes + Evidence full text			

Protein-Protein Interaction (PPI)		IAS(ACT) abstract	ACT full text	ACT abstract
	
		IMS(IMT) full text		IMT full text
	
		IPS(IPT) full text	IPT full text	
	
		ISS full text		

Interactive Annotation Task (IAT) demo				Online GN and gene ranking full text
	
				Online retrieval full text

This special issue contains overview papers for the three tasks, GN [15], PPI [16], and IAT [17]; individual team papers from teams with high impact systems from the GN and PPI tasks; and a paper describing important aspects of text mining from the database curator perspective [18].

## Results

### BioCreative III gene normalization task

The gene normalization (GN) task in BioCreative III was organized by Zhiyong Lu and John Wilbur from the National Center for Biotechnology Information (NCBI). A total of 13 teams participated in the task and submitted 36 official runs. The task required systems to automatically identify genes or gene products mentioned in the literature and link them to EntrezGene database identifiers. This year’s task was a continuation of past GN tasks in BioCreative I and II but with some new features. In terms of the task itself, there were two differences compared to past GN tasks: 1) full text articles were used instead of abstracts; and 2) instead of focusing on specific species (e.g. human in BioCreative II), all species were included in the analysis and no species information was provided. Both changes were implemented to make the GN task closer to a real literature curation task. Indeed, six teams used their GN systems as support for their participation in the realistic curation tasks of the IAT challenge.

Methods used by participants in the current GN task, relied heavily on gene mention finding algorithms developed for past competitions and most of this year’s effort was spent on researching ways to reliably determine the species corresponding to a gene mention. While a number of methods were tried, top performance went to a team that used an information retrieval approach to rank the candidate ids (species). See the GN Overview paper [15] for further discussion on methods.

In addition to the more realistic task, there were two innovative changes to the task evaluation. First, the organizers implemented a novel EM (expectation maximization) algorithm for inferring ground truth based on team submissions and showed its ability to detect differences in team performance. For a discussion of this approach see the GN Overview article [15]. Second, to better measure the quality of rankings in submitted results, a new metric called Threshold Average Precision (TAP-k) [19] replaced the traditional measures (precision, recall, and F-measure) in this year’s task. The TAP-k is a truncated form of mean average precision that truncates the calculation of average precision essentially after seeing *k* irrelevant retrievals. Thus the TAP-k is always lower than the mean average precision and the TAP-k is progressively lower as *k* gets smaller.

In order for teams to optimize their GN systems, the organizers provided two sets of training data consisting of 32 fully annotated full text articles and 500 full text articles annotated only for the genes judged most important for the article, respectively. The test data consisted of 507 full text articles where 50 articles were fully annotated by human curators. The annotations of the remaining 457 articles were inferred by the EM algorithm based on submitted team results. The highest TAP scores (k=5) were 0.3297 and 0.4873 on human-curated and algorithm-inferred annotations, respectively. Compared with results from past GN tasks, the team performance in this year’s challenge is overall lower (see GN Overview paper [15] for discussion of this issue), which can be attributed to the added complexity of full text and the necessity of species identification. By combining team results in an ensemble system, an increased performance of 0.3614 (TAP-5) on the human-curated data was obtained.

### BioCreative III protein-protein interaction task

The PPI task was organized by Martin Krallinger, Florian Leitner, Miguel Vazquez and Alfonso Valencia from the Spanish National Cancer Research Centre in collaboration with the MINT and BioGRID protein interaction databases. This task was inspired directly by the needs of biologists and database curators and structured based on general steps underlying the PPI annotation workflow. The PPI tasks covered 1) the selection of relevant articles (title and abstract) from PubMed (Article Classification Task - ACT); and 2) linking of full text articles to concepts from an ontology that covers terms related to important experimental methods, i.e. interaction detection methods (Interaction Method Task - IMT).

To build systems for the ACT, participating teams were provided with a training set of 2,280 abstracts and a development set of 4,000 abstracts, while the evaluation was carried out on a test set of 6,000 abstracts through comparison to manual labels generated by domain experts. We measured the performance of ten participating teams in this task for a total of 52 runs. The highest (Matthew’s Correlation Coefficient) MCC score measured was 0.55 at an accuracy of 89%, and the best AUC iP/R (interpolated area under the precision/recall curve) was 68%.

In case of the IMT, a total of eight teams submitted 42 runs for a test set of 305 full text articles, out of which 222 were annotation relevant. To implement their systems, teams were provided with a training set of 2,035 and a development set of 587 full text articles. Annotations for the test data set, consisting of associations of full text articles to interaction detection method terms, were generated by curators from the BioGRID and MINT databases. The highest AUC iP/R achieved by any run was 53%, and the best MCC score 0.55. In case of competitive systems with an acceptable recall (above 35%), the macro-averaged precision ranged between 50% and 80%, with a maximum F-Score of 55%.

### BioCreative III interactive task

The interactive task (IAT) in Biocreative III was a demonstration task and was organized by Cecilia Arighi and Cathy Wu from the University of Delaware, and Lynette Hirschman from the MITRE Corporation. The IAT is a special new feature of BioCreative III, designed to address the utility and usability of text mining tools for biocuration and biological knowledge discovery. The aim of this task is to provide the component modules for text mining services to support biocuration as well as general research. In particular, it aims to support real-life tasks by combining multiple text mining tasks to retrieve literature and extract relevant information, and provide results that can be integrated into the curation workflow. This new task complements the others by introducing the development of a user-interface to address the tasks interactively.

A critical aspect of the BioCreative III evaluations is the active involvement of the end users to guide the development and evaluation of useful tools and standards. For this purpose, the User Advisory Group (UAG) was set up, including representatives from model organism databases, protein and protein-protein interaction databases and industry. This group met monthly over a period of nine months with the purpose of defining an appropriate task, gathering system requirements, reaching agreement on various curation issues by working on common examples, testing the systems and providing feedback to the developers.

To encourage participation, the IAT task was built on the GN task, but with the addition of gene ranking (based on the overall importance of the gene in the article) and gene-oriented article retrieval (identifying papers relevant to a selected gene). Six teams participated in this task. A questionnaire was developed to assess the systems. This included informal assessment of the usability (e.g., Is the interface user-friendly?) and the quality (e.g., Is the gene ranking correct?). Each UAG member was assigned a system and two articles to curate which were new to them. Each system was inspected by at least two curators, and a total of four articles were analyzed. The articles were selected based on articles that are problematic for curation, such as those with gene name ambiguity, multiple species, or description of a new gene.

The UAG found the interfaces were generally appealing and easy to use. However, the performance varied substantially from system to system. Some of the problems observed mirrored those of the GN task, for example, in the difficulty of identifying the organism source of a gene. It is widely recognized that text mining is error-prone due to the complexity and ambiguity of natural language. However, an interactive system has the advantage of offering ways to facilitate annotation, such as filtering results by species, allowing the addition or deletion of a species or a gene name, and pointing to contextual information. The UAG concluded that the systems were still at a preliminary stage and needed to improve or add some of these features, and especially to make better use of contextual information.

The retrieval task was not thoroughly assessed mainly due to problems found associated with the gene normalization task: inaccurate species assignments and unresolved name ambiguities, which led to the retrieval of many irrelevant articles. For example, using Entrez :7454 for human WASP, most of the systems returned articles for both WASP and N-WASP, and some systems were not able to discern between WASP as a gene and wasp as an organism, indicating the importance of capturing contextual information.

A demo session during the workshop facilitated face to face communication between developers and curators, and subsequently many suggestions were promptly implemented by the system developers.

## Discussion

For a detailed discussion of the methods applied and results found for the GN [15], PPI [16], and IAT [17] tasks we refer the reader to the individual overview papers dealing with those tasks in this issue. Here we will confine our discussion to what we believe are the important practical implications of results from the BioCreative Workshops and how we believe they should shape future efforts.

### Limitations of current methods

The most important and fundamental goal of the BioCreative Workshops is to provide practical aid to the investigator or curator in dealing with the literature. The first question that seems relevant to this goal is: how accurate are the computer methods currently in use when applied to the BioCreative shared tasks? The article classification (ACT) task for PPI appeared in BioCreative II, II.5, and III. In BioCreative II the task was to select the curatable articles for protein-protein interactions based on the content of the corresponding PubMed abstracts, with testing on a balanced set of equal numbers of positive and negative articles. The highest F score achieved was 0.78 [20]. In BioCreative II.5 the task required the treatment of full text articles and testing involved 595 FEBS Letters articles with 63 positives; the highest F score achieved was 0.85. In BioCreative III testing was on 6000 abstracts from a variety of journals with 15% positives and the best F score was 0.61. The use of abstracts only, unbalanced data, and a wide mixture of journals makes this latter test the most difficult and perhaps the most realistic and highlights the difficulty of a realistic task.

The GN task appeared in BioCreative I, II, and III, with a similar task for protein normalization in BioCreative II.5. In BioCreative I and II it was limited to particular organisms and the best F scores were on fly (0.82), mouse (0.79), and yeast (0.92) in I, and on human (0.81) in II. These results were all obtained on PubMed abstracts. In BioCreative II.5 a similar task (INT) focused on protein normalization with no restriction on organisms; this task required the annotation of full text articles with UniProt IDs for proteins that had experimental PPI evidence in the article. The best raw F score was 0.28 when macro-averaged over all articles [21]. It is important to note that the main difference from the GN tasks was an exhaustive annotation of all genes in the GN tasks and the limitation to proteins with experimental evidence of interaction in the interacting protein normalization task, making the latter significantly more difficult. In BioCreative III the gene normalization task was broadened to involve all organisms and used full text, with the best (break even) F score of 0.50. As a final example, the PPI Interacting Pair Task (IPT) in BioCreative II and II.5 required annotation of an interaction *relation* between pairs of interacting proteins from the full text. In BioCreative II the highest macro-averaged F score was 0.29, while in BioCreative II.5 the highest macro-averaged F score was 0.22. These results show that the shared tasks are very challenging. We believe progress has been made on all these tasks, but it is difficult to quantify the progress because as the tasks have been repeated, they have also become more realistic and hence more difficult.

### The practical use of current methods

Going forward, the questions are how to use the computer algorithms developed and how to determine whether they can indeed enhance the user experience. Let us first ask how the computational methods might be used. In their discussion of the results of the ACT task in BioCreative III, Krallinger, Vazquez, Leitner, and Valencia [22] state that the best results are not of sufficient quality to use as an entirely automated process for curation (at least 43% of relevant documents are missed). The other tasks just mentioned (GN, PPI IPT) in their most realistic form have even lower F scores and a similar concern applies. The opinion was also expressed by Cohen and Hersh [23] that accuracy is not yet sufficient for algorithms performing such tasks to be useful without human interaction. They therefore suggest that algorithms for entity recognition and normalization as well as protein-protein interaction may realistically be used to aid a human curator or investigator. This opinion was later confirmed by the joint results of the FEBS Letters experiment on Structured Digital Abstracts in conjunction with BioCreative II.5, where curator results were compared to author annotations of their own papers and annotations from automated systems [21]. Rebholz-Schuhmann [23] has also noted that biologists “want to read the scientific text to make up their minds what the text conveys” and Peitsch [23] suggests computer-assisted reading as one application of text mining. We also believe, in light of the limited accuracy of current algorithms, that one practical application of text mining will be to aid the investigator in reading or browsing the text of scientific papers. The key point here is that the user will have access to the whole paper and will be able to read at his/her discretion, but the system will help focus attention, help disambiguate expressions, and supply information from external sources as needed.

### A possible use scenario

We turn now to the second question: can we use the methods of entity and relational recognition and normalization, as studied in the BioCreative tasks, to enhance the user’s experience? Will these methods aid in focusing attention, disambiguating expressions, and supplying external information so that the user is benefited in time and effort saved? While we cannot give a definitive answer to this question, we believe the evidence favours a positive answer. First, let us consider the article classification task (ACT). We know that the ACT for PPI is not sufficiently accurate to provide definitive answers on its own. On the other hand, if PPI abstracts were selected manually from a ranked list of automatically generated results, the best system reports half of all relevant abstracts (295) in the top 7% of its result list (421/6000), which translates to a false positive rate of three in ten articles (calculated from the interpolated Precision/Recall curve evaluation for ACT). Given the top ranked 7% of abstracts returned by the system, the user then must decide which are valuable for PPI extraction. Either the user can read the abstracts or alternatively the system could supply an analysis on which its recommendation was based as evidence to the user. The most useful evidence in this regard would be evidence that can be easily and quickly examined by a human to check its validity. To fully implement such an approach will require a refinement of the ACT with a focus on designing systems that can produce such evidence. The goal would be a system that generally supplies easily usable evidence, but which may fail some percentage of the time, with the result that the human has to make a more detailed examination of the document in question. If we are dealing with the use case projected here where only 30% of the abstracts are irrelevant to PPI, then one might argue that supplying such evidence is less important, but we believe such evidence could potentially save the user significant reading time. On the other hand there are situations where a more exhaustive search is in order and it would be essential to examine many more documents with a much higher false positive rate. For example an exhaustive effort could be required to protect a large investment in drug development. It is in such situations that we believe a system that could present easily useable evidence could be most valuable, as an alternative to the user reading the whole abstract. A goal of future BioCreatives will be to perform experiments to measure time saved through the use of automated document ranking enhanced by evidence summaries.

Now suppose one has arrived at a document that gives promise of having curatable PPIs. Let us consider first the GN task for this document. Current GN systems could propose a ranked list of gene identifiers for the article and show the relationship of each identifier to a proposed named entity occurrence in the article. However, we know that the results would not be very accurate. We believe a more useful approach would make the evidence available to the user for each ranked item. A gene/protein ID would be accompanied by the gene mention, the species name, and clickable links into the text where these entities may be found and examined along with their relationship in the text. One may also provide other links to outside information, when available, where the gene name and species name are further described. If one requires high recall, the approach just outlined may not prove to be efficient. As an alternative one can imagine a process which moves linearly through the article highlighting genes/proteins, displaying their database IDs, and allowing the user to either confirm a given entity designation or click on it to obtain further information, potentially leading to a correction of the system output. Such further information could take many forms, from highlighting the near occurrences in the text of species or gene/protein names relevant to the entity in question, to displaying some schematic of the reasoning used by the system to make its initial designation, to providing access to database entries relevant in determining the entity type. Clearly the suggested approaches are only a small part of what is possible.

In any approach the user will be involved in providing corrections to the system and there is also the potential to learn from this feedback in an online fashion to improve subsequent suggestions by the system for a given article and across articles. This feature would allow BioCreative tasks to be designed to be general and species-independent, to support the needs across the larger curation community and address important shared problems, such as gene normalization. For example, even though the Model Organism Database (MOD) curation tasks have many similarities, they still differ in many details, mainly due to the nature of the different organisms under study. The 13 UAG members, who included representatives from different MODs, had lengthy discussions on what makes a gene *primary/curatable* in the literature. Although a final consensus was reached for the IAT task, different views expressed during the formulation of this definition made it clear that each curation group has their own standards and needs for GN. In other words, the definition of “important” genes for the IAT task is likely not to be the same as what a specific MOD decides in practice. Therefore, a useful feature of the GN systems would be to automatically learn the new set of criteria and subsequently re-rank its gene results through interaction with MOD curators.

Finally, suppose the annotation of PPI pairs is the designated task for an article. Then what we have said regarding the GN task is relevant, but just for those gene/protein pairs involved in a PPI. One can still imagine moving linearly through the article examining highlighted gene/protein names, but now ignoring those not involved in a PPI. In addition to highlighting potential gene/protein names, the system can also highlight clues to the existence of a PPI. Again such clues can be clickable to access information about their origin and validity. Though the PPI task has an added level of complexity over the GN task, the same principle applies. In an error prone process the system needs to provide the evidence for its suggestions where possible and in a form as easy to comprehend as possible. In the foregoing discussion we have envisioned a scenario where we believe computational algorithms can provide the most practical impact in assisting a human investigator to interact with the literature in a curation task. The automatic processing of text does not lose any of its importance and we should continue seeking ways to improve algorithms. However, there is recognition that our automatic methods fall short and that from a practical point of view they must provide the evidence for their suggestions and that this evidence must be understandable by a human. Here it must be admitted that current machine learning technology is largely based on weighting many features (often thousands to millions) and it is frequently a challenge to know why a particular recommendation is made. However progress has been made in explaining such opaque models as neural networks and support vector machines [24,25] and this challenge must be dealt with successfully. To the extent that this is successful we believe the algorithms can have a positive impact on the process.

An important lesson learned from the IAT task was that providing specifications of a desired system is not enough; developers and users should team up early on and work together throughout the process of system development. In addition, the user adoption of automated tools into their curation process will depend heavily on performance: systems with many highly ranked incorrect suggestions are not acceptable. Adoption also will depend on the overall convenience of a tool. For example, tools which make use of the synergy of finding the link between a gene mention, its species and its database identifier simultaneously are preferable to tools dealing with species mentions, gene mentions, and subsequently gene ids in separate stages. These observations will guide the development of future BioCreative interactive challenges.

## Conclusions

The goal of the BioCreative Workshops is to promote the development of text mining tools of practical use to database curators and working biologists. Here we have presented a brief history of the BioCreative Workshops and their relationship to other text mining efforts in biology and summarized the results from the GN, PPI, and IAT tasks for the latest BioCreative III Workshop. We observe that the current state of text mining in the field of biology falls short of producing fully automated annotation tools. While work on the basic technology of named entity and relation identification must continue, in order to become relevant to the user this work must find a way to become convenient and labour saving. To answer this challenge we have engaged a user advisory group and initiated user testing of systems. We have also cast a vision of how we believe progress can be made in system functionality. Our aim is to pursue improvements in both user testing and system functionality in future work.

## List of abbreviations used

ACT: article classification task; AUC iP/R: interpolated area under the precision/recall curve; CALBC: collaborative annotation of a large-scale biomedical corpus; CASP: critical assessment of techniques for protein structure prediction; EM: expectation maximization; GM: gene mention; GN: gene normalization; IAS: interaction article subtask; IAT: interactive task; IMS: interaction methods subtask; IMT: interaction method task; INT: interacting protein normalization; IPS: interaction pairs subtask; IPT: interacting pair task; ISS: interaction sentence subtask; JNLPBA: natural language processing in biomedicine and its applications; KDD-CUP: knowledge discovery and data mining competition; LLL: Learning Language in Logic; MCC: Matthew’s correlation coefficient; MOD: model organism database; MUC: message understanding conferences; PPI: protein-protein interaction; TAP: threshold average precision; TREC: text retrieval conferences; UAG: user advisory group

## Competing interests

The authors declare that they have no competing interests.

## Authors' contributions

This paper was drafted by WJW and edited, read, and approved by all authors.
